# Sustainability risk management: Are Malaysian companies ready?

**DOI:** 10.1016/j.heliyon.2024.e24681

**Published:** 2024-01-18

**Authors:** Shazrul Ekhmar Abdul Razak, Mazlina Mustapha, Sabarina Mohammed Shah, Nor Aziah Abu Kasim

**Affiliations:** a Department of Agribusiness and Bioresource Economics, Faculty of Agriculture, Universiti Putra Malaysia, 43400 UPM Serdang, Malaysia; b School of Business and Economics, Universiti Putra Malaysia, 43400 UPM Serdang, Malaysia

**Keywords:** Sustainability risk management, Sustainability risk, Palm oil industry, Contingency theory

## Abstract

The sustainability issues resulting from Malaysian palm oil production have garnered much interest in the palm oil industry. Consequently, Malaysian palm oil industry is indirectly exposed to sustainability risks, including boycott and reputational and regulatory risks. Hence, the industry encounters intense pressure from numerous stakeholders to address sustainability issues. Prior studies propounded that sustainability risk management (SRM) could minimise the adverse impact of sustainability risks by addressing sustainability issues. Nevertheless, the implementation of sustainability risk management in Malaysia remains low as numerous companies are not ready for it. Drawing on contingency theory, the objective of this study is to investigate the influence of contextual factors that can influence companies' readiness in implementing sustainability risk management. Data was collected through the distribution of questionnaires between July and December 2020. A total of 407 questionnaires were distributed, with a response rate of 29 %. Resultantly, sustainability strategy, business size, top management support, and regulatory pressure positively and significantly influenced sustainability risk management implementation. The findings also expanded the current theoretical knowledge with valuable insights for policymakers regarding the factors influencing to companies’ readiness in implementing SRM.

## 1 Introduction

The extensive usage of palm oil in food-based products for cooking as well as non-food products such as biofuel production, oleochemicals, and cosmetics [1,2] led to an increase in the global production of vegetable oil. On a global scale, vegetable oil production exceeded 200 million metric tonnes in the 2020/2021 crop year, with production of palm oil accounting for 36 % of total production or 74.45 million metric tonnes. Today, more than 150 nations currently import and consume palm oil, which is the largest oil traded globally [3]. As top two producer and exporter of palm oil worldwide, Malaysia accounted for 25.8 % of world palm oil production and 34.3 % of world palm oil exports [4]. Locally, the palm oil industry is the major contributor to the agriculture sector at 37.7 %, which is also the third-leading contributor to Malaysian gross domestic product (GDP). Additionally, this industry contributed RM72.3 billion in total export revenue, which increased by 8.4 % in 2020 compared to RM67.55 billion in 2019 [5]. The palm oil industry is also vital to improving the national socioeconomic condition by creating employment opportunities for individuals in rural areas [6]. Thus, the sustainability of the Malaysian palm oil industry is also crucial to Malaysian economic growth and fulfilling global oil and fat demand.

The sustainability issues arising from palm oil production, such as soil property changes, water and air pollution, greenhouse gas emissions, waste, and social conflict have caused much concern among industry stakeholders [7,8]. These issues adversely affect social, environmental, and ecosystem sustainability. Essentially, companies that do not proactively mitigate sustainability issues may be exposed to high sustainability risks, which hampers organisational viability and survivability [9,10]. Giannakis and Papadopoulos [11] explained that sustainability risks negatively affect organisational survivability without disrupting business operations. The European Union (EU) proposed two resolutions to ban palm oil usage in biofuel programmes and alternative sustainability regulations that must be complied with by exporting countries before entering the EU market [12]. Likewise, the United States prohibited the import of palm oil and its associated products from Malaysia following labour issues [13]. The pressure and demand for sustainable palm production are also intensified by various stakeholders towards addressing sustainability issues, causing it challenging for the palm oil industry to retain a competitive edge without disregarding sustainability considerations.

The recent implementation of sustainability risk management (SRM) is becoming more integral, as conventional risk management could not effectively manage sustainability issues [14,15]. Based on a survey by the World Business Council on Sustainable Development in 2017, 70 % of practitioners highlighted the inadequacy of current risk management strategies in mitigating sustainability issues. The SRM strives to alleviate the adverse impact of sustainability risks resulting from sustainability issues. As such, SRM aims to resolve the negative impacts of sustainability risks on organisational survivability by effectively managing sustainability issues [16,17]. Nonetheless, Abdul Aziz [18] demonstrated that environmentally sensitive companies in Malaysia were in the infancy stage of improving their risk management and are not ready in implementing SRM.

Wijethilake and Lama [19] stated that internal and external contextual factors potentially influence the companies' initiative in managing sustainability issues. Arguably, these contextual factors can influence a company's readiness to implement SRM in managing sustainability issues. Rostamzadeh et al. [20] opined that exposure to sustainability risks differs across companies even in similar industries. For example, companies would experience different contextual factors that influence their readiness for SRM implement. In this vein, the internal and external contextual factors of palm oil mills' operational environment would significantly influence SRM implementation. Although several studies investigated SRM implementation [9,18,19,[21], [22], [23], [24]] relevant literature remained scarce owing to ambiguous empirical evidence on the contextual factors influencing SRM implementation. Furthermore, theoretical development in explicating SRM implementation is pivotal.

The present study aimed to provide concrete evidence on the contextual factors influencing organisational readiness to implement SRM through the contingency theory, which expanded the existing knowledge corpus. Several relevant contextual factors in palm oil mills were incorporated from existing literature. In doing so, one research question is expected to be addressed: What are the contextual factors influencing SRM implementation? A quantitative research design by distributing questionnaires was employed to gain pivotal insights on the contextual factors that would significantly impact companies’ readiness to implement SRM among Malaysian palm oil mills.

Section 2 of this paper presents the theoretical framework and hypothesis development. Section 3 highlights the research methodology, while Section 4 denotes the analyses and findings. Section 5 discusses the study outcomes. Finally, Section 6 concludes the study and presents its limitations and recommendations for future works.

## 2 Literature review

### 2.1 Contingency theory

The contingency approach was initially established via organisation theory in the 1960s, which focused on the impact of contingent variables or contextual factors on organisational structure. The management control system (MCS) is an integral part of organisational structure [25]. Otley [26] stated that “the contingency approach to management accounting is based on the premise that there is no universally appropriate accounting system applicable to all organisations in all circumstances; rather a contingency theory attempts to identify specific aspects of an accounting system that are associated with certain defined circumstances and to demonstrate an appropriate matching”.

Waterhouse and Tiessen [27] highlighted the influence of specific organisational circumstances that influence the appropriate MCS. Specifically, the appropriate MCS depends upon the organisation's external business environment and its internal characteristics [28]. This highlights that the appropriate MCS implementation should consider different contextual factors underpinning the organisations [29]. Hence, the theory contingency theory posited that internal and external contextual factors influence the MCS implementation. This study accepts the notion of contingency theory, in which organisational SRM implementation must align with its contextual factors.

The contemporary business environment requires enterprises to promptly fulfil market demand to gain a competitive advantage for survival. For this reason, business organisations have seen a shift in the implementation of MCSs, to suit the current situation of business world, by moving away from traditional management control systems to an advanced one [30,31]. This may also apply to the implementation of SRM. Employing media to highlight sustainability issues through advancements in information technology [32] and business trends, such as stringent sustainability legislation, higher customer demands for sustainable products, and increasing sustainable awareness [11,33], significantly contributed to the sustainability risks existing in firms [20]. As conventional risk management did not provide adequate information for sustainability risks [9,18,22], companies should implement SRM to support managers in decision-making and develop strategies, tactics, and operational policies to regulate corresponding commercial activities.

Beasley et al.’s [34] examination of the influence of contextual factors and risk management in the banking sector via contingency theory revealed the positive association between risk management implementation and business size, industries, top management support, board independence, and the presence of an auditor and chief risk officer. Similarly, a case study regarding the risk management practice of the Birmingham City Council by Wood [35] revealed that the risk management practice was significantly influenced by contextual factors, namely business size, technology, and central government strategies. Gordon et al. [36], who assessed risk management implementation among UScompanies with the contingency theory, indicated five contextual factors (environmental uncertainty, industry competition, business size, firm complexity, and top management support) that influence risk management.

Paape and Speklé [37] investigated risk management implementation in Dutch companies across various industries. Consistently, risk management implementation was significantly influenced by the regulatory environment, audit committee and chief risk officer, the organisational size, the ownership structure, and the industrial types. In examining the factors influencing the level of integrating carbon risk with risk management, Subramaniam et al. [38] revealed the organisational tendency to incorporate carbon risk into current risk management practices in the presence of a formal carbon strategy and internal audit oversight. Furthermore, top management involvement and sufficient resources in terms of personnel and funds would influence organisational decisions to include carbon risk in risk management practices [38].

In light of the aforementioned literature, empirical outcomes on the influence of contextual factors towards SRM implementation remain scarce. Such scarcity necessitates further examination of the contextual factors influencing SRM implementation. This study expanded the theoretical development of SRM implementation through the contingency theory. Following Chenhall [31], this theory would prove a sound understanding of the accounting phenomenon amidst the lack of relevant theoretical frameworks or studies. Siboro et al. [39] underscored the essentiality of the contingency theory in explaining suitable contingencies compared to effective organisational management strategies. In this vein, the contingency theory will “continue to enrich our understanding of the situational contexts of management accounting practice for decades to come” [40]. Applying this theory in the current work aligns with the emergence of SRM research in Malaysia and worldwide.

Contextual factors of sustainability strategy, business size, top management support, perceived environmental uncertainty, and regulatory pressure were analysed based on the contingency theory to ascertain the influence on companies’ readiness in SRM implementation within a developing economy. The following section presents the rationale for including each study variable.

### 2.2 Hypothesis development

Past studies primarily focused on strategy, business size, audit committee, chief risk officer, board of directors, top management support, industrial types, perceived environmental uncertainty, regulation, and technology to pinpoint the impact on risk management implementation. Nonetheless, the contingency theory posited the MCS would only be appropriate in certain contextual factors for business operations [29,31,41], thus indicating that not all contextual factors identified by previous studies were relevant for the current study. Particularly, the industrial type was not suitable for the current study context, which solely focused on the Malaysian palm oil industry without performing a comparison or exploration between different industries. The installation of technology in palm oil production was related to the business size [42,43], which was assessed by this study. Investigating the application of technology to maximise production, minimise liquid waste, and reduce insect attacks would be redundant in this study. Meanwhile, top management support, which constitutes chief risk officers, board of directors, and audit committees, refers to the provision of necessary resources with the authority and expertise to change the organisational direction [[44], [45], [46], [47]]. Overall, this study analysed the contextual factors of sustainability strategy, business size, top management support, perceived environmental uncertainty, and regulatory pressure in palm oil mills.

#### 2.2.1 Sustainability strategy

Past works primarily utilised Miles and Snow's and Porter's typologies of strategy, which is a key contextual factor for MCS application [29,[48], [49], [50], [51]]. Regardless, these strategies prove inappropriate for sustainability-oriented research [[52], [53], [54]]. The lack of empirical evidence of the sustainability strategy-MCS relationship requires further examination. Sustainability strategy incorporates sustainability developmental principles into organisational strategic planning to (i) reduce the operational impact on economic, environmental, and social sustainability via products, processes, and corporate policies [[55], [56], [57]], (ii) demonstrate the organisational commitment to integrating economic, environmental, and social sustainability elements with business operations, (iii) mitigate sustainability issues, and (iv) fulfil sustainable development expectations [19]. Hence, the contingency theory could explain the influence of the sustainability strategy employed by companies on SRM implementation.

Companies that incorporate sustainability strategies can mitigate the impact of business operations on sustainability issues [58]. Stakeholders, specifically those from environmental groups, are concerned about the sustainability issues resulting from palm oil mill operations and palm oil production [1,59]. Existing literature demonstrates that sustainability issues frequently expose firms to multiple sustainability risks, such as reputational damage, negative media attention, boycott, and deteriorated stakeholder relationships, which would jeopardise the company's outlook [9,18]. Thus, sustainability strategy is pivotal to tackling different sustainability issues [60,61] while indirectly preventing sustainability risk.

According to Wijethilake and Lama [19], organisations that formulate sustainability strategy highlights their commitment to avoiding or minimising sustainability risks. Accordingly, companies which have the sustainability strategy will likely implement the SRM. SRM will empower companies to reorganise or devise their business operations to meet the demand for environmental and social sustainability so as to reduce sustainability issues [10]. As an important control system, SRM assists managers in identifying, assessing, and addressing sustainability risks based on the sustainability strategy [17]. Hence, the implementation of SRM in companies is affected by their sustainability strategy. As such, the first hypothesis was proposed:

H1 There is a positive relationship between the sustainability strategy and SRM implementation.

#### 2.2.2 Business size

Contingent theorists place much emphasis on the business size-organisational structure relationship [36,62]. Contingent theory propounds that business size significantly influences MCS implementation [31,63], with larger enterprises more capable of implementing MCS than smaller ones [64,65]. The underlying factor is that “moving from traditional to more contemporary MCSs requires resources and specialists that are only affordable by large companies” [30]. In addition, large companies are generally more visible and exposed to media attention and stakeholders concerning sustainability [66,67]. Palm oil mills’ sustainability activities are salient owing to sustainability issues [68]. Hence, large palm oil mills would be more inclined to implement SRM to address sustainability issues and reduce the adverse impact of sustainability risks. Unlike smaller companies, a control system is implemented to generate accurate sustainability information and make informed decisions [19,69].

Implementing a system to integrate sustainability as part of companies' activities involves a high investment level that poses a high threshold for small companies [70]. For example, companies require a significant amount of funding to install an alternative system or practise innovation. Funding is also crucial to companies conducting training for employees and employing specialists to operate the unfamiliar system [67]. Thus, more resources in terms of facilities, equipment, funding, or employees, would allow a higher opportunity to implement the MCS[38]. In the palm oil industry, high costs and poor returns on investment are among the factors inhibiting smaller mills from implementing a system to address sustainability issues [43], including SRM implementation. Given the paucity of research on the influence of business size on SRM implementation, this study assessed business size based on palm oil mills’ total production capacity in place of total assets and number of employees. The second hypothesis was proposed based on these arguments:

H2 There is a positive relationship between the business size and SRM implementation.

#### 2.2.3 Top management support

The extent to which a company is genuinely interested in sustainability development depends on top management support [19]. Despite the presence of a sustainability strategy, top management support is a key internal factor for sustainability activities [71]. As the direction towards sustainability would systematically and synergistically promote organisational structural change [19,72], top management support is key to successfully implementing sustainability initiatives [56]. Under the contingency theory, the successful implementation of structural change requires the support of top management, who are empowered to develop and implement transformations [73]. Therefore, top management support is considered a strong contextual factor contributing to MCSimplementation within the company [74]. Furthermore, top management is required to prioritise and fulfil stakeholders’ demand for sustainability by integrating sustainability into company activities [19,74] and implement an effective MCS to effectively control sustainability issues [19,21]. The MCS may communicate vital sustainability information to the top management in informing stakeholders, regulating sustainability activities, and providing employees with adequate sustainability training [75].

The SRM plays an integral role in managing sustainability activities to mitigate sustainability issues and risks [19] and the adverse impact of sustainability risks [21]. Palm oil mills receive higher pressure from stakeholders to manage the sustainability issues of mill operations. Accordingly, top management support is crucial to implementing SRM in ensuring the intended company direction and channelling sufficient resources towards managing sustainability issues. Nevertheless, previous studies primarily focused on public-listed companies and large manufacturing companies. The significant findings in the existing studies resulted from generally adequate top management support for SRM implementation in large companies with numerous resources [76]. As such, this study highlighted the impact of top management support on SRM implementation at the business unit level to elevate organisational members’ awareness of sustainability risks. The third hypothesis was proposed:

H3 There is a positive relationship between top management support and SRM implementation.

#### 2.2.4 Perceived environmental uncertainty (PEU)

Environmental uncertainty is a key contextual factor influencing MCS implementation under the contingency theory [29,31,49]. A high environmental uncertainty level would negatively impact company's competitive advantages due to the low possibility of performing informed decisions to respond to the uncertainty [41,62]. Therefore, MCSs are increasingly crucial to generating ample information in encountering an uncertain and unpredictable environment [67]. In a highly uncertain environment, broad coverage of information generated from MCSs would be pivotal to companies in improving decision quality while minimising uncertainty, as the information may provide more potential solutions [73,77].

The significant demand for sustainability compels companies worldwide to respond by integrating sustainability into respective operations. In addition, the rapid shift and development of sustainability, such as consumer preferences, environmental challenges, regulatory changes, and competitor desires elevate the difficulty level of responding to and predicting the environment [75,78]. Existing studies demonstrated firms could implement a MCS amidst high levels of environmental uncertainty to swiftly respond to high sustainability demand [79,80,81]. Enterprises should also develop pertinent strategies for effectively coping with uncertainties when the environment for sustainability is volatile [73]. Hence, information from MCSs may assist enterprises in understanding the uncertain environment [58]. In addition, the need for a MCS is vital to improving communication and coordinating all aspects of the company towards achieving sustainability objectives in an ambiguous environment [78].

The factors influencing palm oil mills' uncertainty level, such as regulatory alterations, importers’ actions, and price fluctuations [[82], [83], [84]] necessitate optimal MCS to reduce the negative impacts. As a tool to address and predict current and future sustainability demands, SRM [14] can fulfil market and stakeholder expectations for sustainable development [19]. Thus, implementing SRM is vital to reducing environmental uncertainty by providing adequate information to avoid the potential sustainability risks arising from commercial activities [38,77]. The PEU impact on the MCS was extensively appraised by previous studies, which indicated the significance of the contextual factor in contingency theory. The essentiality of SRM implementation in expanding current literature on risk management based on the contingency theory led to the fourth hypothesis:

H4 There is a positive relationship between perceived environmental uncertainty and SRM implementation.

#### 2.2.5 Regulatory pressure

Regulatory pressure plays a critical role in promoting the implementation of alternative organisational practices and structures when companies are required to alter existing processes and procedures to comply with compulsory regulations exerted by the government, policymakers, and authorised bodies [85]. Specifically, regulatory pressure motivates MCS implementation [73,86,87] to address the intense demand from consumers and environmental groups for firms to incorporate more sustainability practices [67,88]. Although certain companies employ reactive and proactive approaches in implementing MCSs, different approaches do not lead to the same positive results in addressing sustainability issues [80,89]. Thus, it brings the role of regulatory pressure to implement SRM as MCS, as postulated by contingency theory [73].

Regulatory pressure compels companies to operate under standardised regulations [85] through strict regulation and enforcement, which significantly affect companies’ sustainability structure and behaviour [90]. Sustainability practices through MCS implementation could be motivated by governmental pressure and several influential parties [91]. Therefore, companies must unconditionally adhere to the stipulated regulations and policies [73,92] and operate according to regulations issued by the government, authorised bodies, or policymakers. Regulatory pressure could also be applied through incentive mechanisms, wherein enterprises would gain incentives, such as tax rebates, subsidies, and financial support, for various sustainability efforts, including implementing MCSs [58,67].

The SRM could delineate how companies control organisational activities [11,19,21]. In Malaysia, palm oil mill operations are heavily criticised for the sustainability issues caused by palm oil production [59,68]. The SRM would be an appropriate MCS to control palm oil mill activities and resolve sustainability issues. Nonetheless, SRM remains relatively new to Malaysian companies [18]. Regulatory pressure is crucial for implementing new MCS, where companies are at infancy stage of implementing it [85]. Therefore, regulatory pressure would catalyse the success of implementing SRM in Malaysian palm oil mills, particularly in addressing sustainability issues. In this vein, the fifth hypothesis was proposed:

H5 There is a positive relationship between regulatory pressure and SRM implementation.

## 3 Research method

This quantitative work used a survey questionnaire aimed to investigate the contextual factors influencing companies' readiness to implement SRM. Seven academics and three palm oil mill experts validated the research instrument with a pre-test. The Ethics Committee of Human Research validated and approved the study questionnaire and methodology. Each palm oil mill was contacted through a telephone call to explain the study purpose and seek consent before distributing the questionnaire. A signed cover letter and a questionnaire were emailed to respondents upon receiving permission. Meanwhile, a cover letter highlighting the study purpose, the confidentiality of responses, ethical compliance, voluntary participation, and other crucial details affecting participation, was also distributed together with the questionnaire via email for palm oil mills could not be reached via telephone. Essentially, the individuals’ participation consent reflected the questionnaire completion rate. This questionnaire contains three sections: Section A elicits respondent demographic details; Section B assesses the contextual factors; Section C evaluates SRM variables with established measurement items.

### 3.1 Measurement of constructs

A 7-point Likert scale ranging was used in the survey questionnaire. Four items on sustainability strategy were adapted from Christ and Burrit [81], which were originally extracted from Banerjee et al. [93], to indicate the degree to which sustainability concerns were integrated with the organisational strategic plan. Business size was evaluated through mill production capacity. The gathered data were then converted logarithmically to correct excessive positive skew and increase distribution normality. Five items on top management support were adapted from Wang et al. [92], which was initially extracted from Baird et al. [44], Colwell and Joshi [45], and Banerjee et al. [93] to indicate the degree to which top management provides full support, demonstrates sustainability behaviour, communicates effectively, and reflects adequate sustainability knowledge. Meanwhile, seven items on PEU were adapted from Pondeville et al. [80] to identify the uncertainty level of sustainability rules and regulations, legislation and politics, market demand, competitors’ actions, substitute products, and green competition.

Five items on regulatory pressure were adapted from Jalaludin et al. [91]. Past works determined the government, financial institutions, management, and the market as sources of regulatory pressure. Furthermore, SRM implementation was assessed by 42 items, which were adapted from relevant literature on MCS, risk management, and sustainability. Thirty-six items served to investigate the SRM components, such as risk identification, risk assessment (severity), risk assessment (occurrence), risk assessment (detectability), risk response, and risk monitoring. These items were adapted from Abdullah et al. [7], Giannakis and Papadopoulos [11], Anderson and Anderson [22], Hofmann et al. [32], Abdullah et al. [68], and Jamaludin et al. [94] and divided into economic issues (13 items), environmental issues (13 items), and social issues (10 items). The remaining six items were adapted from Fan et al. [46] to measure risk monitoring. Respondents were asked to indicate sustainability issues, evaluate the severity level, measure the occurrence probability, and determine ease of detection. Additionally, the individuals had to identify risk response strategies (avoidance, control, retention, and sharing) to resolve sustainability issues and ascertain the risk monitoring implementation level to regulate palm oil mills’ risk management process.

### 3.2 Sample and data collection

Malaysian palm oil mills were selected as study samples based on the responsibility level for producing crude palm oil (CPO): the main oil palm unit used for cooking, food processing, oleo cosmetics, and biofuel programmes. The Malaysian palm oil industry is export-oriented, with India and China being the two key importers. The total export of CPO alone exceeds 70 % of the overall export of palm oil products. This percentage substantially contributes to the total export revenue, which boosts the economic growth of the palm oil industry. The significance of CPO production, export performance, and export revenue performance in the local agricultural sector, national economy, and socio-economy [95] denotes the importance of palm oil mills. A directory issued by the Malaysian Palm Oil Board (MPOB) was used to derive information on palm oil mills, including names, addresses, telephone numbers, and email addresses.

The MPOB is a government agency under the Ministry of Plantation Industries and Commodities that governs the palm oil industry developments. Essentially, this regulatory body promotes and develops national sustainability objectives, policies, and programmes for industrial viability. The MPOB is also authorised to licence, regulate, and coordinate palm oil industry activities, such as the issuance of risk management practices: Hazard and Critical Control Points (HACCP), Environmental Impact Assessment (EIA), and Social Impact Assessment (SIA) requirements. All industry stakeholders must adhere to these regulations.

As of December 31, 2019, 457 palm oil mills were registered under the 2020 MPOB directory. Fifty of them were excluded following the pilot test, thus resulting in the recruitment of 407 palm oil mills. Past research disclosed a low response rate from an emerging accounting issue in Malaysia [91]. As such, the current sample size from the overall population was determined via the census approach to improve the response rate. Mill managers, assistant managers, supervisors, engineers, executives, safety officers, sustainability officers, and other employees with direct involvement in palm oil production were chosen as target respondents. These individuals were recruited based on their role in ensuring sustainable palm oil production via industrial standards compliance to avoid potential issues. Summarily, the respondents' position, experience, and knowledge are pivotal to providing reliable information to assess the sample mills’ operational performance.

### 3.3 Response rate

Notably, 407 questionnaires were distributed to all Malaysian palm oil mills from July to December 2020. A total of 121 responses yielded an initial response rate of 29.7 %. Three incomplete questionnaires were discarded during the data cleaning stage, which elicited a final response rate of 29 %. The response rate was deemed satisfactory compared to those reported in risk management, environmental management accounting, and sustainability studies [38,80]. Generally, the response rate for a Malaysian study that employs survey questionnaires ranges between 20 % and 30 % [[96], [97], [98]]. A non-response bias test was conducted to determine notable variances between the response and non-response groups. The former and latter were represented by the early and late response groups, respectively [99]. As bias can influence the variable interpretations and overall data analysis outcomes, it is deemed vital to conduct a non-response bias test. This study conducted the non-response bias test following Oppenheim's [100] recommendation. The first 30 respondents in the early response group were compared against the last 30 counterparts in the late response group to generate accurate outcomes. Notably, some sustainability and environmental accounting studies have used this non-response bias testing approach. The first and last 30 respondents were extracted to represent early and late response groups, respectively, as variances with close proximity may instigate biased analysis [58]. The independent *t*-test outcomes implied no significant difference between individuals who responded early compared to those who responded late. In other words, no non-response bias was identified in this study.

#### 3.3.1 Respondent demographics

The respondents’ various roles are presented in Table 1. Mill managers comprised 33.9 % of the respondents, followed by assistant managers (22 %), engineers (20.3 %), safety, sustainability, and compliance officers (11 %), administrative officers (8.5 %), and other employees with direct involvement in palm oil mill operations (4.2 %). In terms of tenure, more than 45 % of the respondents worked for over six years, 30.5 % of them worked for three to five years, and 20.3 % worked for one to three years. A total of 22 % of respondents worked for six to 10 years, with 7.6 % of them having over 10 years of experience. All the respondents reflected sufficient knowledge and experience in palm oil mill operations and high data reliability.

**Table 1:** Respondent demographics.

Description	Frequency (n = 118)	Percentage (%)
**Position**		
Manager	40	33.9
Assistant Manager	26	22.0
Engineer	24	20.3
Safety, Sustainability, and Compliance Officer	13	11.0
Administrative Officer	10	8.5
Others	5	4.2
*Total*	*118*	*100*
**Tenure in the Current Position**		
1–3 years	40	33.9
3–5 years	43	36.4
6–10 years	26	22.0
More than 10 years	9	7.6
*Total*	*118*	*100*
**Tenure in the Palm Oil Industry**		
1–3 years	24	20.3
3–5 years	36	30.5
6–10 years	26	22.0
More than 10 years	32	27.1
*Total*	*118*	*100*
**State**		
West Malaysia/Peninsular Malaysia		
*Southern Region*	16	13.6
*Central Region*	12	10.1
*Northern Region*	17	14.4
*East Coast Region*	31	26.3
East Malaysia/Borneo Island		
*Sabah*	29	24.6
*Sarawak*	13	11.0
*Total*	*118*	*100.0*
**Mill Ownership**		
Independent-Owned	31	26.3
Government-Owned	37	31.4
Private-Owned	50	42.3
*Total*	*118*	*100.0*
**Mill Establishment**		
Less than 5 years	28	23.7
5–10 years	11	9.3
11–15 years	11	9.3
More than 15 years	68	57.6
*Total*	*118*	*100*
**Mill Production (Tonne/Hour)**		
Less than 30 tonnes per hour	9	7.6
30–60 tonnes per hour	91	77.1
More Than 60 tonnes per hour	18	15.3
*Total*	*118*	*100*

With regards to the palm oil industry, 35.6 % of the 118 sample palm oil mills were located in East Malaysia or Borneo Island. The remaining 64.4 % were located in West Malaysia. Furthermore, 42.3 % of the mills were private, 31.4 % were government-owned, and 26.3 % were independent-owned. Over 65 % of these mills have operated for more than a decade. Concerning palm oil production, 15.3 % of the mills produced over 60 metric tonnes per hour, with 77.1 % generating 30–60 tonnes per hour and 7.6 % generating under 30 tonnes per hour.

## 4 Analysis and findings

Partial least squares structural equation modelling (PLS-SEM) was used in this study to analyse multiple variables and indicators and a complex framework [101,102]. Specifically, this approach proved suitable for assessing the research framework with lower-order constructs (LOCs) and high-order constructs (HOCs) [103] and small sample sizes. Comprising six variables and over 50 items, this study indicated the complexity of the research framework. The current work encompassed one HOC (SRM implementation). In terms of sample size, the 118 valid data were deemed inappropriate to employ covariance-based structural equation modelling (CB-SEM) following the prerequisite for a large sample size. The PLS-SEM, which entailed a measurement and structural model, was the most appropriate data analysis technique for this study.

### 4.1 Measurement model

In this study, LOCs and HOCs comprised the measurement model. The LOCs with reflective indicators implied contextual factors. As such, the construct meaning would not be altered with the deletion of an indicator. Contrarily, omitting any of the six formative dimensions of SRM implementation would change the meaning. Each dimension encompassed highly correlated and interchangeable indicators. Specifically, SRM implementation was categorised as a Type II reflective-formative HOC [104]. A two-stage method was employed to specify and estimate the HOCs. The first stage only measured the LOCs in the study model, which were directly correlated to constructs with theoretical connections to the HOCs. Meanwhile, the second stage used the LOCs' latent variable scores to measure the HOCs. Internal consistency reliability, convergent validity, and discriminant validity tests were conducted to validate the measurement model of LOCs. Table 2 presents the LOCs' internal reliability and convergent validity. Notably, sustainability strategy, business size, top management support, risk identification, risk assessment and analysis (occurrence), risk response, and risk monitoring attained satisfactory threshold values of 0.7, 0.7, and 0.5 for factor loadings and Cronbach's alpha, composite reliability (CR), and average variance extracted (AVE), respectively. The constructs satisfied the internal consistency and convergent validity requirements.

**Table 2:** Internal reliability and convergent validity.

Construct	Indicator	Loading	Cronbach's Alpha	CR	AVE
Sustainability Strategy (SS)	SS1	0.808	0.854	0.892	0.674
	SS2	0.863			
	SS3	0.803			
	SS4	0.808			
Business Size (BS)	BS	1.000	1.000	1.000	1.000
Top Management Support (TMS)	TMS1	0.877	0.922	0.940	0.759
	TMS2	0.871			
	TMS3	0.898			
	TMS4	0.825			
	TMS5	0.883			
Perceived Environmental Uncertainty (PEU)	PEU2	0.655	0.808	0.860	0.513
	PEU3	0.571			
	PEU4	0.555			
	PEU5	0.759			
	PEU6	0.830			
	PEU7	0.865			
Regulatory Pressure (RP)	RP2	0.624	0.724	0.828	0.549
	RP3	0.813			
	RP4	0.828			
	RP5	0.680			
Risk Identification (RI)	RIECON	0.742	0.775	0.871	0.693
	RIENV	0.891			
	RISOC	0.856			
Risk Assessment and Analysis-Severity (RAS)	RASECON	0.513	0.763	0.855	0.677
	RASENV	0.962			
	RASSOC	0.919			
Risk Assessment and Analysis-Occurrence (RAO)	RAOECON	0.696	0.839	0.901	0.756
	RAOENV	0.949			
	RAOSOC	0.940			
Risk Assessment and Analysis-Detectability (RAD)	RADECON	0.632	0.831	0.889	0.733
	RADENV	0.939			
	RADSOC	0.959			
Risk Response (RR)	RR	1.000	1.000	1.000	1.000
Risk Monitoring (RM)	RM1	0.910	0.946	0.957	0.790
	RM2	0.941			
	RM3	0.931			
	RM4	0.765			
	RM5	0.889			
	RM6	0.887			

*Note:* PEU1 and RP1 were deleted.

Although Cronbach's alpha, CR, and AVE values of PEU, regulatory pressure, risk assessment and analysis (severity), and risk assessment and analysis (detectability) exceeded the minimum threshold, the indicators revealed factor loadings below 0.7. Byrne [105] denoted that indicators with a loading equal to or exceeding 0.5 are adequate if the construct's AVE value represented by the indicators is above 0.5. The current study constructs achieved an AVE value exceeding 0.5 and fulfilled internal and convergent validity. Following the small sample size that deterred the PLS algorithm from conducting the analysis, risk identification, risk assessment and analysis (severity), risk assessment and analysis (occurrence), and risk assessment and analysis (detectability) were measured via item parcelling. All the construct indicators were aggregated into economic (ECON), environmental (ENV), and social (SOC) issues, which functioned as indicators for the four constructs.

The Forner-Lacker criterion and HTMT ratio were used to check discriminant validity. Based on Table 3, the AVE value of a construct proved higher than the squared correlation between the construct and all other constructs. This outcome implies adequate discriminant validity. Following Table 4, all the constructs attained an HTMT value below the HTMT_.85_ value of 0.85 or the HTMT_.90_ value of 0.90 [106]. As such, no discriminant validity issues were detected. The HOCs' measurement model was also validated by measuring collinearity issues and analysing the formative indicators’ significance and relevance [107]. The variance inflation factor (VIF) served to identify collinearity issues. The VIF values in Table 5 were under the threshold value of 10 [108]. Hence, collinearity issues were not detected in this study. The relevance of formative indicators was also examined. Excluding risk assessment (occurrence), risk assessment (detectability), and risk monitoring, all the formative indicators attained significant outer weights. Non-significant indicators were removed upon determining the outer loadings. Essentially, significant formative indicators with outer loadings exceeding 0.5 were retained [107]. Risk assessment (occurrence), risk assessment (detectability), and risk monitoring achieved outer loadings above 0.5 and proved significant. Summarily, all the formative indicators were deemed significant and relevant.

**Table 3:** Discriminant validity through the forner-lacker criterion.

	SS	BS	TMS	PEU	RP	RI	RAS	RAO	RAD	RR	RM
SS	0.821										
BS	0.096	1.000									
TMS	0.692	0.079	0.871								
PEU	0.166	−0.114	0.208	0.716							
RP	0.181	−0.184	0.036	−0.024	0.741						
RI	−0.013	−0.164	0.053	0.265	0.081	0.832					
RAS	0.048	−0.164	0.162	0.250	0.080	0.505	0.823				
RAO	−0.087	−0.193	0.008	0.149	0.051	0.504	0.599	0.870			
RAD	−0.230	−0.154	−0.109	0.052	0.024	0.448	0.472	0.762	0.856		
RR	−0.216	0.098	−0.189	0.000	0.089	−0.049	−0.165	−0.227	−0.156	1.000	
RM	−0.032	0.045	−0.081	−0.018	−0.229	−0.128	−0.116	−0.030	0.028	−0.024	0.889

**Table 4:** Discriminant validity through the HTMT ratio.

	SS	BS	TMS	PEU	RP	RI	RAS	RAO	RAD	RR	RM
SS											
BS	0.094										
TMS	0.779	0.105									
PEU	0.242	0.127	0.228								
RP	0.293	0.203	0.198	0.174							
RI	0.091	0.186	0.074	0.317	0.107						
RAS	0.080	0.161	0.173	0.293	0.119	0.677					
RAO	0.111	0.207	0.094	0.18	0.073	0.628	0.752				
RAD	0.215	0.163	0.13	0.154	0.062	0.575	0.553	0.817			
RR	0.203	0.098	0.18	0.081	0.114	0.056	0.162	0.252	0.160		
RM	0.065	0.049	0.078	0.146	0.266	0.148	0.124	0.061	0.078	0.066

**Table 5:** Collinearity issues, outer weights, and outer loadings.

HOC	LOCs	VIF	Outer Weight	t-value	p-value	Outer Loading	p-value
SRM	RI	2.453	−0.394	2.832	0.005	0.416	0.003
	RAS	3.863	0.385	2.227	0.026	0.825	0.000
	RAO	4.756	0.133	0.679	0.497	0.836	0.000
	RAD	4.278	0.279	1.458	0.145	0.752	0.000
	RR	6.360	0.553	2.130	0.033	0.945	0.000
	RM	6.457	0.003	0.013	0.990	0.897	0.000

### 4.2 Structural model

Based on the R-square (*R*^*2*^) of the research model (0.497), 49.7 % of SRM implementation was explained by exogenous variables (contextual factors), which denoted a robust research model following Ramayah et al. [109] (see Table 6). The predictive relevance (*Q*^*2*^) values for all the constructs derived from blindfolding exceeded the value of 0. Hence, the study model attained adequate predictive relevance. Effect sizes (ƒ^2^) served to evaluate the relative impact of the predictor constructs on the dependent variable. Table 6 demonstrates that top management support produced a medium effect (ƒ^2^ = 0.113) on SRM implementation. Sustainability strategy (ƒ^2^ = 0.062), business size (ƒ^2^ = 0.031), and regulatory pressure (ƒ^2^ = 0.035) produced a small effect while PEU (ƒ^2^ = 0.004) exerted no effect on SRM implementation. Furthermore, Table 6 depicts that sustainability strategy and business size positively and significantly influenced SRM implementation (*p* < 0.05), which supports H1 and H2. Top management support also significantly and positively influenced SRM implementation (*p* < 0.001), which supports H3. In addition, regulatory pressure significantly influenced SRM implementation (*p* < 0.05), thus supporting H5. Nevertheless, PEU did not significantly influence SRM implementation, hence not supporting H4.

**Table 6:** Structural model results and hypothesis testing.

Hypothesis	Std. Beta	Std. Error	t-value	p-value	VIF	*R*^*2*^	*ƒ*^*2*^	*Q*^*2*^
H1: SS → SRM	0.264	0.101	2.600	0.009 **	2.247	0.497	0.062	0.444
H2: BS → SRM	0.129	0.058	2.249	0.025 **	1.074		0.031	
H3: TMS → SRM	0.344	0.096	3.569	0.000 *	2.082		0.113	
H4: PEU → SRM	0.047	0.058	0.808	0.419	1.082		0.004	
H5: RP → SRM	0.170	0.083	2.050	0.040 **	1.640		0.035	

*Notes: t* > 1.96; *p < 0.001; **p < 0.05 (two-tailed).

## 5 Discussion

Based on the research findings, sustainability strategy, business size, top management support, and regulatory pressure significantly and positively influenced SRM implementation in palm oil mills. The findings provided valuable insights into the limited literature on SRM implementation, particularly in developing countries. Specifically, sustainability strategy played a significant role in SRM implementation owing to the unique nature of the palm oil industry governed by the MPOB [6]. The MPOB acts as the main policymaker in issuing sustainability guidelines, policies, and practices for all industry players, including palm oil mills. In terms of the business environment, the result postulated that an effectively implemented and established sustainability strategy enabled palm oil mills to pursue sustainability practices as part of daily operations, thus reducing sustainability issues. Palm oil mills would implement SRM to assist the operations in achieving sustainability objectives when being committed to addressing sustainability issues. The current study outcomes revealed that MCS implementation complements particular strategies, enhancing the notion of contingency theory in risk management. As sustainability objectives are becoming increasingly important, the growing need for sustainability strategies is a crucial contextual factor in influencing the implementation of MCS. Consistent with contingency theory, the findings indicated that a well-formulated and established sustainability strategy is highly vital to companies’ readiness in implementing SRM. In other words, companies are required to implement an appropriate MCSto support respective sustainability strategy [75].

The findings revealed that larger palm oil mills were more inclined to implement SRM. One of the reasons could be that larger palm oil mills in Malaysia have more capital to employ savvier technology [83]. Hence, they could do the same by utilising more resources in implementing SRM to control operations in addressing sustainability issues. In addition, large palm oil mills are generally more environmentally visible and subject to greater public scrutiny. For instance, Cargill Inc. and Unilever Global switched to other palm oil suppliers after learning that IOI Corporation encountered sustainability issues [59]. Larger mills with sufficient resources to recruit specialists or train current employees understand the nature of sustainability issues and are more prepared to implement SRM while increasing public confidence. Such local mills operate on large scales to lower production costs and increase profits [42]. Following the contingency theory, implementing MCS is contingent upon the organisational context. A palm oil mill's size depends on its production capacity. Consequently, organisational context significantly influenced SRM implementation. Business size plays a pivotal role in influencing companies' readiness to implement SRM.

The essentiality of top management support in MCSwas highlighted in terms of green management practices, environmental protection behaviour [45], and environmental management accounting (EMA) adoption [65,73]. By underscoring the critical role played by top management support in SRM implementation, the empirical outcomes expanded the current body of literature on risk management. Malaysian palm oil mills’ SRM implementation could be facilitated with strong top management support. The SRM implementation enables companies to minimise sustainability issues while optimising sustainable development for sustainable palm oil production. Top management who are committed to sustainable palm oil production are more predisposed to implement SRM to monitor mill operational activities upon realising the potential benefits of implementing SRM. The implementation of SRM would be more effortless with top management support and required resources, such as funding, specialists, and techniques [92]. The findings enriched the contingency theory by indicating that SRM would be implemented and effective in the presence of top management. Hence, top management support is vital in driving any changes in business practices or new implementation of MCSsuch as SRM, which leads to higher company readiness.

No evidence was discovered for PEU to significantly influence SRM implementation, which could be due to the highly regulated palm oil industry in Malaysia [6]. Specifically, the operations of palm oil mills are well-structured, ranging from the collection of FFBs to sterilisation, stripping, digestion and pressing, clarification, purification, drying, and storage [68], focusing on producing sustainable crude palm oil in accordance with sustainability requirements. Due to being highly regulated and well-structured, Razak et al. [110] found that sustainability issues are moderately easy to predict. Hence, the unpredictable sustainability risk arising from sustainability issues claimed by Giannakis and Papadopoulos [11] was not exemplified in Malaysian palm oil mills. Perceivably, local palm oil mill managers did not rely on MCS to improve managerial decision-making and mitigate sustainability issues under certain conditions or in a stable environment. As such, the insignificant finding enriched the contingency theory, where higher environmental uncertainty was associated with a higher attachment to MCSs in producing relevant information. Although the findings contradicted previous studies, Malaysian companies are relatively not ready to implement an alternative MCS, including SRM, when the business environment is stable.

The findings demonstrated that high pressure from regulators would compel palm oil mills to implement SRM as a controlling system, which paralleled prior studies on the impact of regulatory pressure and several MCSs, such as EMA [91,92], environmental MCSs [80], carbon management accounting [77], and carbon risk management [38]. Regulatory pressure, which primarily originates from regulations and enforcement, is a vital contextual factor to influence SRM implementation when Malaysian firms, particularly palm oil mills, are in the preliminary stage of implementation. The Malaysian palm oil industry is highly regulated, with industry players, including palm oil mills, required to adhere to over 15 environmental laws and regulations [94]. The significant impact of regulatory pressure also posited that palm oil mill activities were subject to further examination through governmental sustainability policies owing to the strong law enforcement by the ministry and authorities. High regulatory pressure for managing sustainability issues would elevate the companies’ readiness to implement SRM.

## 6 Conclusion

The current study examined the contextual factors influencing companies’ readiness to implement SRM through the contingency theory, namely sustainability strategy, business size, top management support, perceived environmental uncertainty, and regulatory pressure. Resultantly, sustainability strategy, business size, top management support, and regulatory pressure positively and significantly influenced SRM implementation. Meanwhile, perceived environmental uncertainty did not significantly influence SRM implementation. The findings supported the existing argument, in which the contextual factors of a company would influence organisational readiness to implement SRM in managing sustainability issues. Specifically, the readiness of Malaysian palm oil mills in implementing SRM is influenced by sustainability strategy, business size, top management support, perceived environmental uncertainty, and regulatory pressure.

Several theoretical and practical implications were yielded from the study outcomes, which provided alternative evidence of the relationships between contextual factors and SRM implementation and enriched the contingency theory. Based on the study outcomes, contextual factors play a vital role in determining when a specific MCS is appropriate for organisations in a specific situation. These results expanded the current body of knowledge on sustainability, risk management, and management accounting based on the contingency theory. Such operationalisation could provide a sound understanding of the significant impacts of internal and external contextual factors on SRM implementation, with different types of contextual factors yielding varied responses. Theoretically, this study provided sufficient empirical evidence to delineate the notable influence of a company's internal and external contextual factors on the organisational readiness to incorporate SRM. Particularly, top management support was the major internal factor, while regulatory pressure was the driving external factor based on the effect size, which generated additional insight into the existing literature that internal factors (top management support) and external factors (regulatory pressure) were significant in implementing MCSs. Other internal and external factors, namely sustainability strategy, business size, and perceived environmental uncertainty, produced a lower influence or an insignificant impact on SRM implementation. Nevertheless, the findings demonstrated that internal and external factors of an organisation could elucidate the rationales for employing different systems to suit different organisational requirements, as postulated by contingency theory.

Practically, the finding acknowledged the importance of a formulated and standardised strategy for sustainability-related initiatives. The sustainability strategy formulated by the MPOB also enabled palm oil mills to direct resources towards managing sustainability issues to fulfil stakeholders' demand for sustainable palm oil production. Since sustainable development is a collective approach, the study findings served as a benchmark for policymakers to provide more capital and resources for SRM implementation among small palm oil mills as a stepping stone to elevate their readiness. Traditionally, sustainability issues were externalised to the natural environment and society [11] before the growing consumer awareness of sustainability issues demanded enterprises to internalise sustainability issues [19,21]. Top management possesses major responsibility for investing in a system to maintain optimal relationships with stakeholders and obtain legitimacy and reputation. Palm oil companies and policymakers can internalise the significance of top management support in promoting and implementing SRM. In addition, the findings underscored policymakers' pivotal role in instilling regulatory pressure to promote the benefits of implementing SRM in palm oil mills, which influence companies’ readiness. Therefore, the findings provide useful insights for palm oil mills as well as companies in Malaysia to comprehend the drivers for the successful implementation of SRM. Specifically, the findings highlight that companies in Malaysia need to consider different contextual factors, ranging from the external business environment to internal characteristics, for the implementation of SRM.

The present findings reflected several limitations, which provided future research avenues. A quantitative design was employed to collect data through a structured closed-ended questionnaire, wherein the findings could be limited by the survey method. Respondents could only select pre-determined responses without opportunities for other possible responses. Furthermore, the survey was distributed to the respondents through postage and email without the presence of a researcher, which might render inaccuracy or misinterpretation of the question meaning. Future researchers are recommended to also employ qualitative research through a series of in-depth interviews to obtain detailed information and develop a deeper understanding of the factors contributing to SRM implementation. The study data was solely derived from palm oil mills in Malaysia, which could reduce the outcome generalisability to other industry players or environmentally sensitive companies. Future research could replicate this study across different settings to gauge the applicability of the current findings to the global context apart from industry-specific characteristics. Additionally, the study variables were only applied to the current palm oil industry. Other unexamined factors deriving from other theoretical frameworks such as organisational resources and capabilities, technological development, industrial characteristics, institutional pressure, and national policy might be relevant to shaping the company perception of and response to sustainability risk. Future researchers could appraise different settings when selecting pertinent variables for research framework development.

## CRediT authorship contribution statement

**Shazrul Ekhmar Abdul Razak:** Writing – review & editing, Writing – original draft, Methodology, Formal analysis, Data curation. **Mazlina Mustapha:** Writing – review & editing, Writing – original draft, Supervision, Funding acquisition, Formal analysis. **Sabarina Mohammed Shah:** Supervision, Formal analysis, Conceptualization. **Nor Aziah Abu Kasim:** Supervision, Formal analysis, Conceptualization.

## Discussion and conclusion – Shazrul Ekhmar Abdul Razak

### Funding disclosure

This research work is funded by 10.13039/501100004530UPM 10.13039/501100003782IPS GRANT (GP-10.13039/501100003782IPS/2020/9682900).

## Declaration of competing interest

The authors declare that they have no known competing financial interests or personal relationships that could have appeared to influence the work reported in this paper.

## References

[1] Lim C.I., Biswas W., Samyudia Y. (2015). Review of existing sustainability assessment methods for Malaysian palm oil production. Procedia CIRP.

[2] Naidu L., Moorthy R. (2021). A review of key sustainability issues in Malaysian palm oil industry. Sustainability.

[3] Mielke T. (2017). Encyclopedia of Sustainability Science and Technology.

[4] MPOC (2021). MPOC.

[5] Nordin I., Hassan Z., Razali N.A.M. (2021). http://mpoc.org.my/malaysian-palm-oil-sector-performance-in-2020-and-market-opportunities/.

[6] Begum H., Alam A.S.A.F., Awang A.H. (2019). Sustainability of Malaysian oil palm: a critical review. Int. J. Environ. Sustain Dev..

[7] Abdullah I., Mahmood W.H.W., Fauadi H.F.M., Rahman M.N.A., Mohamed S.B. (2017). Sustainability manufacturing practices in Malaysian palm oil mills: priority and current performance. J. Manuf. Technol. Manag..

[8] Naidu L., Moorthy R., Huda M.I.M. (2023). The environmental and health sustainability challenges of Malaysian palm oil in the European union. Journal of Oil Palm Research.

[9] Wong A. (2014). Corporate sustainability through non-financial risk management. Corp. Govern..

[10] Singh K., Abraham R., Yadav J., Agrawal A.K., Kolar P. (2023). Linking CSR and organizational performance: the intervening role of sustainability risk management and organizational reputation. Soc. Responsib. J..

[11] Giannakis M., Papadopoulos T. (2016). Supply chain sustainability: a risk management approach. Int. J. Prod. Econ..

[12] Kushairi A., Ong-Abdullah M., Nambiappan B., Hishamuddin E., Zainal Bidin M.N.I., Ghazali R., Subramaniam V., Sundram S., Parveez G.K.A. (2019). Oil palm economic performance in Malaysia and R&D progress in 2018. Journal of Oil Palm Research.

[13] MPOC (2018). http://www.mpoc.org.my/The_implications_of_EU_Resolution_to_the_Malaysian_Palm_Oil_Industry.aspx.

[14] Abdul Aziz N.A., Abdul Manab N., Othman S.N. (2016). Critical success factors of sustainability risk management (SRM) practices in Malaysian environmentally sensitive industries. Procedia - Social and Behavioral Sciences.

[15] Haywood L.K. (2022). Putting risk management into the corporate sustainability context. Soc. Responsib. J..

[16] Abdul Aziz N.A., Manab N.A., Othman S.N. (2015). Exploring the perspectives of corporate governance and theories on sustainability risk management (SRM). Asian Econ. Financ. Rev..

[17] Valinejad F., Rahmani D. (2018). Sustainability risk management in the supply chain of telecommunication companies: a case study. J. Clean. Prod..

[18] Abdul Aziz N.A., Abdul Manab N., Othman S.N. (2016). Sustainability risk management (SRM): an extension of enterprise risk management (ERM) concept. Int. J. Manag. Sustain..

[19] Wijethilake C., Lama T. (2018). Sustainability core values and sustainability risk management: moderating effects of top management commitment and stakeholder pressure. Bus. Strat. Environ..

[20] Rostamzadeh R., Ghorabaee M.K., Govindan K., Esmaeili A., Nobar H.B.K. (2018). Evaluation of sustainable supply chain risk management using an integrated fuzzy TOPSIS- CRITIC approach. J. Clean. Prod..

[21] Abdul Aziz N.A., Abdul Manab N., Othman S.N. (2016). Managing the adverse impact of crises and disasters through sustainability risk management (SRM). Science International Journal.

[22] Anderson D.R., Anderson K.E. (2009). Sustainability risk management. Risk Manag. Insur. Rev..

[23] Soomro M.A., Lai F.-W. (2017). Examining a new paradigm of enterprise sustainability risk management. Global Business and Management Research: Int. J..

[24] Yilmaz A.K., Flouris T. (2010). Managing corporate sustainability : risk management process based perspective. Afr. J. Bus. Manag..

[25] Zuriekat Majdy Issa Khalil (2005). http://eprints.hud.ac.uk/4613/.

[26] Otley D. (1999). Performance management: a framework for management control systems research. Manag. Account. Res..

[27] Waterhouse J.H., Tiessen P. (1978). A contingency framework for management accounting systems research. Account. Org. Soc..

[28] Khan M.H.U.Z., Ahmed R., Halabi A.K. (2010).

[29] Gosselin M. (2011). Contextual factors affecting the deployment of innovative performance measurement systems. J. Appl. Account. Res..

[30] Abdel-Kader M., Luther R. (2008). British Accounting Review.

[31] Chenhall R.H. (2003). Management control systems design within its organizational context: findings from contingency-based research and directions for the future. Account. Org. Soc..

[32] Hofmann H., Busse C., Bode C., Henke M. (2014). Sustainability-related supply chain risks: conceptualization and management. Bus. Strat. Environ..

[33] Zimmer K., Fröhling M., Breun P., Schultmann F. (2017). Assessing social risks of global supply chains: a quantitative analytical approach and its application to supplier selection in the German automotive industry. J. Clean. Prod..

[34] Beasley M.S., Clune R., Hermanson D.R. (2005). Enterprise risk management: an empirical analysis of factors associated with the extent of implementation. J. Account. Publ. Pol..

[35] Woods M. (2009). A contingency theory perspective on the risk management control system within Birmingham City Council. Manag. Account. Res..

[36] Gordon L.A., Loeb M.P., Tseng C.Y. (2009). Enterprise risk management and firm performance: a contingency perspective. J. Account. Publ. Pol..

[37] Paape L., Speklé R.F. (2012).

[38] Subramaniam N., Wahyuni D., Cooper B.J., Leung P., Wines G. (2015). Integration of carbon risks and opportunities in enterprise risk management systems: evidence from Australian firms. J. Clean. Prod..

[39] Siboro D.T., Siahaan A.M., Muda I., Ginting S. (2018).

[40] Ashton D., Beattie V., Broadbent J., Brooks C., Draper P., Ezzamel M., Gwilliam D., Hodgkinson R., Hoskin K., Pope P., Stark A. (2009). British research in accounting and finance (2001-2007): the 2008 research assessment exercise. Br. Account. Rev..

[41] Khan H., Ahmed R., Halabi A.K., Tsamenyi M., Uddin S. (2010).

[42] Azman I. (2014). The impact of palm oil mills' capacity on technical efficiency of palm oil millers in Malaysia. Oil Palm Industry Economic Journal.

[43] Loh S.K., Nasrin A.B., Mohamad Azri S., Bukhari N.A., Muzammil N., Stasha Elanor R.A. (2019). Biogas capturing facilities in palm oil mills: current status and way forward. Palm Oil Engineering Buletin.

[44] Baird K., Harrison G., Reeve R. (2007).

[45] Colwell S.R., Joshi A.W. (2013). Corporate ecological responsiveness: antecedent effects of institutional pressure and top management commitment and their impact on organizational performance. Bus. Strat. Environ..

[46] Fan H., Li G., Sun H., Cheng T.C.E. (2017). An information processing perspective on supply chain risk management: antecedents, mechanism, and consequences. Int. J. Prod. Econ..

[47] Meiryani (2014). Influence of top management support on the quality of accounting information system and its impact on the quality of accounting information. Res. J. Finance Account..

[48] Govindarajan V. (1988). A contingency approach to strategy implementation at the business-unit level: integrating administrative mechanisms with strategy. Acad. Manag. J..

[49] Hoque Z. (2004). A contingency model of the association between strategy , environmental uncertainty and performance measurement : impact on organizational performance. International business review.

[50] Khani A., Ahmadi M. (2012). Performance measurement using balanced scorecard measures and strategy based on Miles a nd Snow ’ s typology in Iran. Afr. J. Bus. Manag..

[51] Miles R.W., Snow C.C. (1978).

[52] Ferreira A., Moulang C., Hendro B. (2010). Environmental management accounting and innovation: an exploratory analysis. Account Audit. Account. J..

[53] Magsi H.B., Ong T.S., Ho J.A., Hassan A.F.S. (2018). Organizational culture and environmental performance. Sustainability.

[54] Park W., Sung C.S., Byun C.G. (2019). Impact of unlisted small and medium-sized enterprises' business strategies on future performance and growth sustainability. Journal of Open Innovation: Technology, Market, and Complexity.

[55] Baumgartner R.J., Rauter R. (2017). Strategic perspectives of corporate sustainability management to develop a sustainable organization. J. Clean. Prod..

[56] Engert S., Baumgartner R.J. (2016). Corporate sustainability strategy - bridging the gap between formulation and implementation. J. Clean. Prod..

[57] Saunila M., Nasiri M., Ukko J., Rantala T. (2019). Smart technologies and corporate sustainability: the mediation effect of corporate sustainability strategy. Comput. Ind..

[58] Latan H., Jabbour C.J.C., de Sousa Jabbour A.B. L., Wamba S.F., Shahbaz M. (2018). Effects of environmental strategy, environmental uncertainty and top management’s commitment on corporate environmental performance: The role of environmental management accounting. J. Clean. Prod..

[59] Hafizuddin-Syah B.A.M., Shahida S., Fuad S.H. (2018). Sustainability certifications and financial profitability: an analysis on palm oil companies in Malaysia. Jurnal Pengurusan.

[60] Begum H., Alam A.S.A.F., Er A.C., Abdul Ghani A.B. (2019). Environmental sustainability practices among palm oil millers. Clean Technol. Environ. Policy.

[61] Choong C.G., Mckay A. (2014). Sustainability in the Malaysian palm oil industry. J. Clean. Prod..

[62] Jusoh R. (2010). The influence of perceived environmental uncertainty , firm size , and strategy on multiple performance measures usage. Afr. J. Bus. Manag..

[63] Hoque Z., James W. (2000). Linking balanced scorecard measures to size and market factors: impact on organizational performance. J. Manag. Account. Res..

[64] Passetti E., Tenucci A. (2016). Eco-efficiency measurement and the in fluence of organisational factors : evidence from large Italian companies. J. Clean. Prod..

[65] Phan T.N., Baird K., Su S. (2017). The use and effectiveness of environmental management accounting. Australas. J. Environ. Manag..

[66] Welbeck E.E., Owusu G.M.Y., Bekoe R.A., Kusi J.A. (2017). Determinants of environmental disclosures of listed firms in Ghana. International Journal of Corporate Social Responsibility.

[67] Mokhtar N., Jusoh R., Zulkifli N. (2016). Corporate characteristics and environmental management accounting (EMA) implementation : evidence from Malaysian public listed companies (PLCs). Journal of Cleaner Production Journal.

[68] Abdullah I., Wan Mahmood W.H., Fauadi M.H.F.M., Rahman M.N.A., Ahmad F. (2015). Sustainability in Malaysian palm oil: a review on manufacturing perspective. Pol. J. Environ. Stud..

[69] Teh B.H., Ong T.S., Jaffar N., Masoudi S.Y.S.A. (2015). Sustainable performance measurement (SPMs) model: effects of product tecnology and process technology. Pertanika Journal of Social Sciences and Humanities.

[70] Álvarez Jaramillo J., Zartha Sossa J.W., Orozco Mendoza G.L. (2019). Barriers to sustainability for small and medium enterprises in the framework of sustainable development—literature review. Bus. Strat. Environ..

[71] Foerstl K., Reuter C., Hartmann E., Blome C. (2010). Journal of Purchasing & Supply Management Managing supplier sustainability risks in a dynamically changing environment — sustainable supplier management in the chemical industry. J. Purch. Supply Manag..

[72] Kasim A. (2015). Environmental management system. Int. J. Contemp. Hospit. Manag..

[73] Ong T.S., Teh B.H., Selley S., Magsi H. (2018). The relationship between contingent factors that influence the environmental management accounting and environmental performance among manufacturing companies in Klang Valley, Malaysia. International Journal of Economics and Management.

[74] Zahid M., Rahman H.U., Muneer S., Butt B.Z., Isah-Chikaji A., Memon M.A. (2019). Nexus between government initiatives, integrated strategies, internal factors and corporate sustainability practices in Malaysia. J. Clean. Prod..

[75] Wijethilake C. (2017). Proactive sustainability strategy and corporate sustainability performance: the mediating effect of sustainability control systems. J. Environ. Manag..

[76] Kitsis A.M., Chen I.J. (2021). Do stakeholder pressures influence green supply chain Practices?Exploring the mediating role of top management commitment. J. Clean. Prod..

[77] Kumarasiri J., Gunasekarage A. (2017). Risk regulation, community pressure and the use of management accounting in managing climate change risk: Australian evidence. Br. Account. Rev..

[78] Maletič M., Maletič D., Gomišček B. (2018). The role of contingency factors on the relationship between sustainability practices and organizational performance. J. Clean. Prod..

[79] Appiah B.K., Donghui Z., Majumder S.C., Monaheng M.P. (2020). Effects of environmental strategy, uncertainty and top management commitment on the environmental performance: role of environmental management accounting and environmental management control system. Int. J. Energy Econ. Pol..

[80] Pondeville S., Swaen V., De Rongé Y. (2013). Environmental management control systems: the role of contextual and strategic factors. Manag. Account. Res..

[81] Christ K.L., Burritt R.L. (2013). Environmental management accounting : the signi fi cance of contingent variables for adoption. J. Clean. Prod..

[82] Isa M.A.M., Baharim A.T., Mohamed S., Noh M.K.A., Nasrul F., Ibrahim W.M.F.W., Hassan S.S. (2020). Crude palm oil price fluctuation in Malaysia. Int. J. Acad. Res. Bus. Soc. Sci..

[83] Norhidayu A., Nur-Syazwani M., Radzil R., Amin I., Balu N. (2017). The production of crude palm oil in Malaysia. International Journal of Economics and Management.

[84] Rahman A.K.A., Balu N., Shariff F.M. (2013). Impact of palm oil supply and demand on palm oil price behaviour. Oil Palm Industry Economic Journal.

[85] Aziz Abdul, Azah Nor, Senik R., Foong S.Y., Ong T.S., Attan H. (2017). Influence of institutional pressures on the adoption of green initiatives. International Journal of Economics and Management.

[86] Christ K.L. (2014). Water management accounting and the wine supply chain : empirical evidence from Australia. Br. Account. Rev..

[87] Muhammad-Jamil C.Z., Mohamed R. (2017). Antecedent factors of environmental management accounting practice. Int. J. Econ. Res..

[88] Maas K., Schaltegger S., Crutzen N. (2016). Integrating corporate sustainability assessment, management accounting, control, and reporting. J. Clean. Prod..

[89] Nwoba A.C., Boso N., Robson M.J. (2021). Corporate sustainability strategies in institutional adversity: antecedent, outcome, and contingency effects. Bus. Strat. Environ..

[90] Kumari S., Patil Y.B. (2019). Enablers of sustainable industrial ecosystem: framework and future research directions. Manag. Environ. Qual. Int. J..

[91] Jalaludin D., Sulaiman M., Nik Ahmad N.N. (2011). Understanding environmental management accounting (EMA) adoption : a new institutional sociology perspective. Soc. Responsib. J..

[92] Wang S., Wang H., Wang J. (2018). Exploring the effects of institutional pressures on the implementation of environmental management accounting: do top management support and perceived benefit work?. Bus. Strat. Environ..

[93] Banerjee S.B., Iyer E.S., Kashyap R.K. (2003).

[94] Jamaludin N.F., Hashim H., Muis Z.A., Zakaria Z.Y., Jusoh M., Yunus A., Abdul Murad S.M. (2018). A sustainability performance assessment framework for palm oil mills. J. Clean. Prod..

[95] Nambiappan B., Ismail A., Hashim N., Ismail N., Shahari D.N., Idris N.A.N., Omar N., Salleh K.M., Hassan N.A.M., Kushairi A. (2018). Malaysia: 100 years of resilient palm oil economic performance. Journal of Oil Palm Research.

[96] Abu Bakar L.J., Ahmad H. (2010). Assessing the relationship between firm resources and product innovation performance: a resource-based view. Bus. Process Manag. J..

[97] Ahmad M.F., Ismail S.N., Hassan M.F., Wei C.S., Abdul N., Ahmad A.N.A., Nawi M.N.M., Rahman N.A.A. (2019). A study of green factory practices in Malaysia manufacturing industry. Int. J. Supply Chain Manag..

[98] June S., Mahmood R. (2011). Role ambiguity and job performance of. Malaysian Management Journal.

[99] Lineback J.F., Thompson K.J. (2010).

[100] Oppenheim A.N. (2001).

[101] Richter N.F., Sinkovics R.R., Ringle C.M., Schlägel C. (2016). A critical look at the use of SEM in international business research. Int. Market. Rev..

[102] Rigdon E.E. (2014). Rethinking partial least squares path modeling: breaking chains and forging ahead. Long. Range Plan..

[103] Hair J.F., Hult G.T.M., Ringle C.M., Sarstedt M. (2017).

[104] Sarstedt M., Hair J.F., Cheah J.H., Becker J.M., Ringle C.M. (2019). How to specify, estimate, and validate higher-order constructs in PLS-SEM. Australas. Market J..

[105] Byrne B.M. (2016). Structural Equation Modeling with AMOS.

[106] Henseler J., Ringle C.M., Sarstedt M. (2015). A new criterion for assessing discriminant validity in variance-based structural equation modeling. J. Acad. Market. Sci..

[107] Hair J.F., Ringle C.M., Sarstedt M. (2011). PLS-SEM: indeed a silver bullet. J. Market. Theor. Pract..

[108] Hair J.F., Black W.C., Rabin B.J., Anderson R.E. (2010).

[109] Ramayah T.J.F.H., Cheah J., Chuah F., Ting H., Memon M.A. (2018).

[110] Razak S.E.A., Mustapha M., Kasim N.A.A., Shah S.M. (2020). Sustainability risk management using failure mode effect analysis: evidence from Malaysia. European Journal of Molecular & Clinical Medicine.

